# SCFAs’ pleiotropic role in pathogenesis and salutogenesis: mechanisms in exacerbation and regulation of inflammation and fibrosis from gut to host

**DOI:** 10.1099/jmm.0.002117

**Published:** 2026-01-29

**Authors:** Saleem Ahmad, Sikander Ali, Asad Ur Rehman, Ikram Ul Haq, Iram Liaqat, Muhammad Nauman Aftab, Tadesse Shume

**Affiliations:** 1 Dr. Ikram-ul-Haq Institute of Industrial Biotechnology, Government College University, Lahore, Pakistan; 2 Department of Zoology, Government College University, Lahore, Pakistan; 3 Department of Microbiology, School of Health Sciences, Haramaya University, Haramaya, Ethiopia

**Keywords:** cytokines, disease, dysbiosis, fibrosis, gut microbiota, health, inflammation, short-chain fatty acids (SCFAs)

## Abstract

Short-chain fatty acids (SCFAs) are essential gut microbiota metabolites with significant effects that are well recognized for their anti-inflammatory benefits, yet their pro-inflammatory and pleiotropic properties have received little attention in literature. SCFAs produced by gut bacteria from one to five carbons engage with a network of G-protein-coupled receptors such as FFAR2/GPR43, FFAR3/GPR41, Olfr78 and monocarboxylate transporters (MCT-1–MCT-4) to influence host physiology. Through established signalling pathways including Mitogen-Activated Protein Kinase (MAPK), mTOR and G*α*i/G*α*q, SCFAs serve as acetyl CoA precursors that facilitate lipogenesis, gluconeogenesis and cholesterol synthesis while also activating NF*κ*B and reactive oxygen species pathways (e.g. succinate), potentially resulting in vascular inflammation. While SCFAs typically suppress inflammation through histone deacetylase inhibition and immune regulation, pro-inflammatory roles emerge in specific settings. Within immune compartments, SCFAs exhibit cell-specific effects, from priming cell-driven pro-inflammatory roles in one type of immune cell to suppression of inflammatory mediators in others. Moreover, SCFAs can lead to fibrotic remodelling, an intensified form of inflammation in both intestinal and distant tissues. This review aims to demonstrate the complex biphasic bridge between aggravation and resolution influenced by factors such as cell type, study methodologies, receptors, dose dependency, age, metabolic changes and inherent properties and concludes with the significance of particular and accurate research approaches to mimic the true environment and observe SCFA effects employing humanized mice, gut-on-chip systems and organoids for more precise and relevant results.

## Graphical Abstract

SCFAs exert dual roles in health and disease by modulating immune responses, inflammation, and fibrosis across organs, influencing salutogenesis and pathogenesis through dose, cell-specific, and contextual factors.

## Introduction

**Fig. 1.**
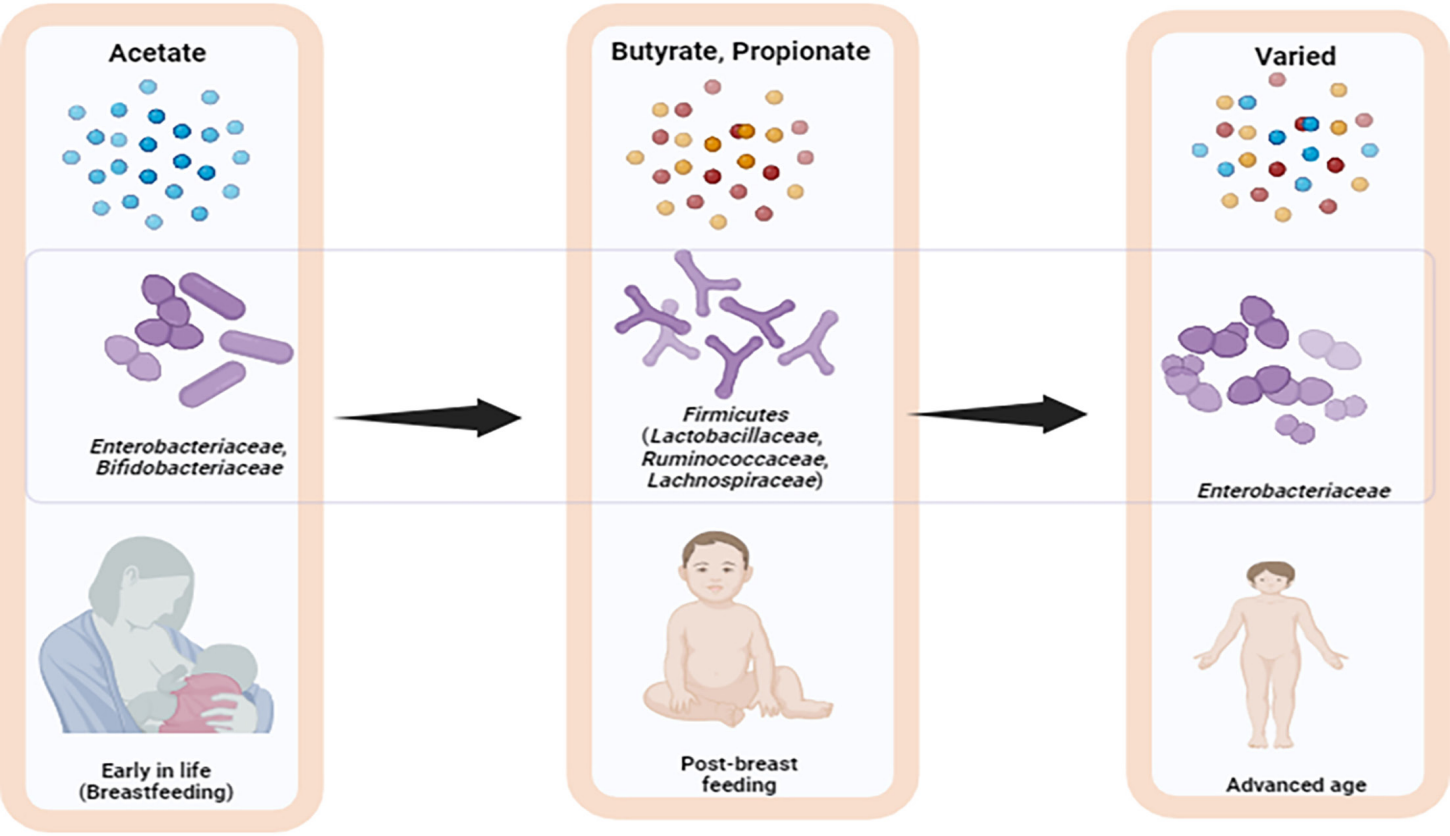
SCFAs composition changes with development along with gut microbiota diversification.

Short-chain fatty acids (SCFAs) are fermentation-derived metabolites produced primarily through the breakdown of non-digestible dietary fibres and polysaccharides by the gut microbiome [1]. SCFAs are organic acid molecules containing carbon chains ranging from C1 to C5 with a carboxylic group attached, playing crucial roles in various processes in the human body, including bioenergetics, homeostasis, immunomodulation, gut defence barrier maintenance and influencing disease and health conditions at both intestinal and extra-intestinal levels [2,4]. The most extensively produced and studied SCFAs are acetate, propionate and butyrate (C2, C3 and C4, respectively), with acetate contributing to nearly three-fifths of the total SCFAs, while propionate and butyrate individually contribute one-fifth [5,6]. Additionally, there are relatively low levels of other fatty acids produced, including branched-chain or isomers such as 2-methyl butyrate, succinate, iso-butyrate and iso-valerate, contributing as little as 5% of the total SCFAs in the gut metabolome [7,8]. As of 2020, a diverse array of 74 bacterial species have been identified and classified into 4 major metabolic pathways – the acetyl-CoA pathway, succinate pathway, acrylate pathway and propanediol pathway – all known for their ability to produce SCFAs [7,10]. A few minor pathways, such as acetogenesis by *Clostridium*, *Eubacterium*, *Acetogenium*, *Butyrbacterium*, *Acetobacterium*, *Acetoanaerobium* and *Pelobacter*; carbon fixation by *Bacteroides succinogenes*, *Clostridium butyricum* and *Syntrophomonas* sp.; and butyrate kinase by *Coprococcus* and specific *Clostridium* spp., also play a role in SCFA production, as reviewed in the literature [1,11, 12]. SCFA synthesis in the human gut is influenced by various factors, including age [13,14], nutrition [15] and the availability of substrates for microbial fermentation [16], highlighting the intricate interplay between host and microbial metabolism that contributes to overall gut health and disease prevention. *Enterobacteriaceae* and *Bifidobacteriaceae* dominate the gut during early development, producing acetate, a precursor to branched-chain fatty acids (BCFAs) [13,14]. After weaning, the gut microbiota shifts to the *Firmicutes* phylum, which produces more butyrate and propionate [17]. In older age, *Enterobacteriaceae* reappear, leading to a more diverse SCFA profile (Fig. 1) . A high-fibre diet promotes SCFA-producing bacteria, while a combination of fibre and a protein-based diet is crucial for gut microbiota composition and metabolic output [18,19].

### Gut–body interaction: transporters, receptors and signalling cascades

Following production in the gut, SCFAs can work in two ways. First, they are absorbed by colonocytes in the gut through specialized transporters, where they play a role in cellular processes and then bypass through the portal blood circulation, reaching the liver and other organs in the body. This emphasizes their critical function in the gut–body axis, which contributes to general health and influences the aetiology of illnesses beyond the gut. Second, SCFAs trigger signalling pathways through receptors expressed on gut epithelial–immune cells and cells in organs such as hepatocytes, kidney cells, adipocytes and cardiovascular epithelial cells, mediating local and systemic effects in inflammation and fibrosis [20].

SCFAs primarily enter the colonic epithelium through carrier-mediated pathways, with monocarboxylate transporter 1 (MCT1) and MCT4 and H+-coupled channels (transporters) playing crucial roles. While MCT1 is located on the apical membrane, both MCT1 and MCT4 are situated on the basolateral membrane of colonic epithelial cells [21]. Concentrations of SCFAs in the start (proximal colon) range from 9 to 131 mmol l^−1^, decreasing to 11–80 mmol l^−1^ in the distal colon. The transport of SCFAs into cells is mainly facilitated by MCT1, MCT4, SMCT1 and SMCT2 transporters [22]. The colonic epithelial cells metabolize the majority of SCFAs, with the remaining amount of SCFAs reaching the superior mesenteric vein, inferior mesenteric vein and portal vein through passive diffusion and active transport via transporters [23]. The concentrations of acetate, propionate and butyrate in these veins are ~262.8, 30.3 and 30.1 µM l^−1^, respectively. While some SCFAs undergo hepatic processing, the rest enter peripheral circulation at levels of 172.9 µM l^−1^ for C2, 3.6 µM l^−1^ for C3 and 7.5 µM l^−1^ for C4 [20,24]. Passive diffusion is another mechanism through which SCFAs secreted by gut microbiota reach gut and systemic organs via blood, although this type of transport is very minute and not significant in playing a major role since SCFAs are present in dissociated form, requiring an active transport mechanism [25].

G-protein-coupled receptors (GPCRs) are one of the most studied and diverse groups of cell membrane receptors [26], with ~800–1,000 genes encoding these receptors in the human genome, ~50% of which are relevant to the pharmaceutical industry [27,28]. GPCRs are classified into six classes (A–F) using the GRAFS system, with the rhodopsin family being the most prominent. GPCRs consist of an N-terminus and a C-terminus in extracellular and intracellular positions, respectively, along with seven transmembrane *α*-helices of varying lengths. The N and C termini are often modified, essential for receptor activity (desensitization and internalization) [29]. GPCRs recognize a wide range of natural ligands, including fatty acids produced by gut microbiota [30]. GPCRs are expressed on almost all cells, including immune cells like macrophages and monocytes [31,32]. Among GPCRs, GPR41, also known as free fatty acid receptor 3 (FFAR3), and GPR43, also known as FFAR2, are of prime interest in the context of this review, as they recognize SCFAs. GPR109A is also a receptor for SCFAs expressed in colonocytes and immune cells and studied to be involved in the anti-inflammatory process [11,33]. GPR41 and 43 have a strong affinity for propionate, while butyrate and acetate bind to GPR41 and GPR43 with tight junctions, respectively [34]. Other GPCRs, such as olfactory receptor-78, known as Olfr78 in mice, the equivalent of which is OR51E2 in humans, are a subgroup of receptors with a strong emphasis on study, notably the former. Olfr78, which primarily binds to acetate and propionate, increases cAMP levels and promotes renin release. This receptor is highly expressed in vascular smooth muscle (VSM) cells of the peripheral vasculature and the renal afferent arterioles, where it plays an important role in controlling vascular tone and kidney function [25,35]. These GPCRs are the main players in shaping the intestinal health–disease scenario, with SCFAs being the pawns in this intricate system.

Recent work has expanded the canonical SCFA receptor landscape beyond FFAR2/3 and GPR109A to include extra-olfactory odorant receptors that respond to microbial SCFAs. In rodents, Olfr78 (and the human ortholog OR51E2) binds acetate and propionate and couples to G-protein signalling pathways that diverge from, yet crosstalk with, canonical SCFA GPCRs [36,37]. Activation of Olfr78 in juxtaglomerular cells and arterioles increases intracellular cAMP via G_s coupling and stimulates renin release, providing a direct mechanistic link between microbial metabolites and the renin–angiotensin axis; this Olfr78-mediated renin release opposes the vasodilatory effects mediated by GPR41 in vascular beds, producing a context-dependent effect of SCFAs on systemic haemodynamics [38]. In human cells, high-resolution structural and functional studies of OR51E2 show that propionate binds the receptor’s orthosteric pocket and promotes G_s recruitment with subsequent increases in cAMP; OR51E2 activation has also been linked to downstream MAPK/extracellular signal-regulated kinase (ERK1/2) signalling in extra-olfactory cells (for example, prostate and immune cells), indicating potential roles in cellular proliferation, inflammatory modulation and metabolic regulation. Collectively, these data indicate that Olfr78/OR51E2 act as bona fide SCFA sensors that engage G_s–cAMP and ERK pathways, influence renin secretion and vascular tone and likely contribute to broader metabolic and immunomodulatory effects of gut microbiota-derived SCFAs [35,39].

#### GPCR-mediated signalling pathways

SCFAs regulate inflammation through a complex network of GPCRs and transporter proteins. This intricate interaction is critical for maintaining immunological homeostasis and regulating inflammatory responses [40]. This dual mechanism of action enables SCFAs to efficiently coordinate metabolic and inflammatory responses, emphasizing their pleiotropic promise in health and disease.

##### MAPK signalling pathway

The c-Jun N-terminal kinases, ERKs and p38 MAPKs are the three subfamilies that make up the MAPK family. Extracellular signals are sent to the nucleus by MAPKs, resulting in the control of gene expression [41]. SCFAs interact with MAPK components in various ways. They inhibit histone deacetylases (HDACs) that deacetylate MKP-1, promoting the release of pro-inflammatory cytokines through MAPK signalling. Examples of these HDACs include HDAC1–3. Additionally, SCFAs activate GPR41 and GPR43 receptors, which leads to the phosphorylation of p38 and ERK1/2, stimulating the synthesis of chemokines and pro-inflammatory factors [42]. However, Trichostatin A (TSA)-induced HDAC inhibition reduces macrophage production of TNF-*α* and IL-6 without altering the phosphorylation of ERK1/2 and p38 [43]. These findings underscore the dual role of SCFAs in modulating pro- and anti-inflammatory cytokine responses through MAPK signalling pathways.

##### mTOR signalling pathway

SCFAs regulate the Mechanistic/mammalian Target of Rapamycin (mTOR) signalling pathway, impacting cellular metabolism and immune responses. Through the activation of mTORC1 and mTORC2 via GPCRs and inhibition of HDACs, SCFAs can stimulate the production of both pro-inflammatory and anti-inflammatory cytokines, thereby playing a pivotal role in maintaining immune homeostasis, cellular growth and inflammation [22,44]. mTOR pathways show divergent outcomes in various cell-dependent milieus vis-à-vis activation by SCFAs. An experimental dataset of Yang and colleagues [45] showed that butyrate activated and enhanced the activity of mTOR and STAT3 (both phosphorylated) after 24 and 6 h of incubation, respectively. Activated by butyrate, mTOR and STAT3 contrastingly regulated the HIF-1*α* and Ahr expression in CD4+ T lymphocytes. HIF-1*α* is found to be bound directly to the IL-22 promoter and upregulates IL-22. Despite the author’s iteration on the protective role during inflammation, IL-22 is more of a two-edged sword which, in addition to anti-inflammatory/healing processes, could lead to very nasty outcomes, including chronic inflammation and even cancer due to any dysregulation [46]. Reactive oxygen species (ROS) production by SCFAs, particularly butyrate, is a well-established phenomenon. A study by Pant *et al.* [47] showed the butyrate-derived ROS-induced inhibition of activation/phosphorylation of mTOR leading to autophagy of hepatic carcinoma cells. On the contrary, as reported by Yan *et al.* [48], butyrate induced enhanced pro-inflammatory cytokine (IL-1*β*, TNF-*α*, NLRP) expression, halted ROS generation and inhibited autophagy in the leukemic monocyte-derived THP-1 cancerous cell line.

##### G*α*i and G*α*q pathway

SCFAs activate GPR43 and GPR41 receptors, leading to the initiation of diverse downstream signalling pathways. When GPR41 is activated, it couples with G*α*i, resulting in the inhibition of adenylyl cyclase activity and a subsequent decrease in cAMP levels [49,50]. On the other hand, activation of the GPR43 receptor by SCFAs involves coupling with both G*α*i and G*α*q, leading to a decrease in cAMP levels and an increase in cytoplasmic calcium release. Activation of G*α*q triggers the stimulation of phospholipase C, which hydrolyses phosphatidylinositol 4,5-bisphosphate into inositol 1,4,5-trisphosphate (IP3) and diacylglycerol, subsequently causing the release of IP3-induced Ca2+ from the endoplasmic reticulum. Meanwhile, G*α*i/o activation inhibits adenylyl cyclase, reducing cAMP levels and activating ERK1/2 through Src proteins. FFAR3, which interacts with G*α*i proteins, follows a similar mechanism to decrease cAMP levels and activate ERK1/2 through PI3K [51,56]. This signalling cascade is crucial for protein kinase C (PKC) activity, characterized by the signalling of pro-inflammatory cytokines, cell proliferation, secretion, barrier function and gene regulation processes. Pro-inflammatory molecules attract other cells such as dendritic cells (DCs), neutrophils and macrophages, leading to the establishment of inflammation [57,59]. GPR receptors influence NF*κ*B signalling by upregulating the *β*-arrestin 2 pathway. GPR43 reduces the nuclear NF*κ*B subunits p65 and p50, thereby decreasing the production of pro-inflammatory cytokines and chemokines, including IL-1*β* and IL-6 [60,61].

## SCFAs: inflammatory triggers in the body

SCFAs such as acetate play a crucial role in lipogenesis and gluconeogenesis in intestinal cells and beyond [62]. Acetate serves as a primary precursor in the biosynthesis of the cholesterol pathway, ultimately leading to cholesterol accumulation [63]. According to a study conducted by Dietschy and McGarry [64], acetate (^14^C radiolabelled) produced a cytosolic acetyl-CoA pool used for the synthesis of cholesterol that was not in isotopic equilibrium with the mitochondrial pool used for ketogenesis. Goodwin and Margolis [65] reported up to 20-fold increased incorporation of radiolabelled acetate into cholesterol upon incubation with mice liver homogenate. A similar approach by Dietschy and Brown [66] indicated that the synthetic rate of cholesterol from acetate varied in a range of up to 100-fold using various altered liver samples. The non-equilibrium relation of acetyl-CoA pool derived from acetate was also found between mitochondria and cytosol. Similarly, infusion of stable SCFAs (C radiolabelled) in the caecum of conscious mice indicated acetate and butyrate were used for palmitate and cholesterol synthesis; propionate was used for glucose production [67]. Butyrate has been reported to accumulate phosphate- and vitamin-induced calcification in VSMs via HDAC inhibition and NF*κ*B activation pathways [68]. The accumulation of cholesterol (LDL) or calcification in blood vessels can cause inflammation of the arteries (as seen in atherosclerosis), with cholesterol’s molecularly modified form in arteries acting as a stimulant for immune cells’ (macrophages) pattern recognition receptors [PRRs, such as toll-like receptors (TLRs)], triggering the formation of a pro-inflammatory cascade. The heightened response of TLR, combined with cholesterol crystals in the cytoplasm, further exacerbates this inflammatory process and results in the release of pro-inflammatory cytokines and chemokines by activating receptors like the NLRP3 family [69,72]. In addition to systemic inflammation, SCFAs have been studied for their potential association with obesity [73,75]. For instance, a study reported by Perry *et al.* [76] demonstrated that high levels of acetate can affect ghrelin hormone secretion, leading to obesity in rats. One of the risk factors linked to obesity is the deposition of fat and cholesterol through diet, all of which can contribute to gut inflammatory diseases like Crohn’s disease (CD) and inflammatory bowel disease (IBD) [77,78].

Although propionate and butyrate have been widely studied and reviewed for their essential role in suppressing both systemic and intestinal inflammation, a reduced or diminished concentration of these SCFAs is a dysbiotic cause of inflammation [79]. Propionate is known to inhibit the conversion of acetate into cholesterol and fatty acids, so its low levels are likely to contribute to inflammation due to increased cholesterol production [20,80]. Similarly, butyrate also enhances the synthesis of long-chain fatty acids [20], which in turn can cause the onset of low-grade inflammatory conditions by modulating several transcriptional factor pathways such as PPAR-*α*/*γ* and NF*κ*B [81].

Succinate is produced by gut microbiota in small quantities as well as derived from metabolic pathways (TCA). Succinate is generally presumed to be associated with its pro-inflammatory role in various metabolic diseases [82]. There is a staggering amount of data filling links among IBD, colitis, CD and the presence of succinate or succinate-producing bacteria [83,86]. After accumulating in macrophages, succinate produces ROS, triggers metabolic reprogramming and causes phenotype shifting, all of which transform macrophages into the M1 or pro-inflammatory type and release a variety of pro-inflammatory mediators [87].

### Pro-inflammatory role of SCFAs in gut–body axis

Leucocytes present in the gut can also exhibit inflammatory signalling pathways when SCFAs activate GPR43 and GPR41. As a result, pro-inflammatory cells with high calcium levels activate PKC, which, along with reduced cAMP due to G*α*i/o protein activation, enhances cell motility and activation by rolling, adhesion and expression. This leads to the release of pro-inflammatory cytokines such as TNF-*α *and IL-1*β*, driving the inflammatory response [49,59, 88]. GPR43 plays a crucial role in neutrophil chemotaxis and pro-inflammatory cytokines. Neutrophils, comprising 60% of white blood cells (WBCs), are involved in establishing the inflammatory response, while cytokines are crucial in maintaining gut-immune homeostasis [89,90]. Neutrophils not only recruit monocytes and DCs, but they also influence macrophage differentiation into the pro-inflammatory type; neutrophil migration is critical for chemokines and integrins. Similarly, studies have shown that GPR43-deficient mice experience more severe colitis symptoms with elevated levels of IL-17, CINC-2*αβ* and TNF-*α*, indicating increased inflammation [59,91]. Despite this, mouse models partially elucidate the precise role of GPR43 and GPR41 in chemokine production through activation of ERK1/2 and p38 MAPK pathways [57], highlighting the need for additional research on humans.

SCFAs produced in abundance are fuel for metabolic machinery in gut epithelia, liver, kidney and other cells. More SCFA production in the gut increases mitochondrial energy metabolism, which in turn increases the generation of ROS via the mitochondrial pathway [92] in gut epithelia as well as neutrophils, which could eventually cause a leucocyte burst [93] through chemotactic receptor signals. Although ROS is short-lived and effectively inactivated by specialized enzymes in an antioxidant process [94], prolonged availability and imbalance impose oxidative stress, leading to various conditions, including intestinal and systemic inflammation [95]. A study by Kumar *et al.* [92] demonstrates that butyrate, an SCFA produced by gut bacteria, influences epithelial signalling through the generation of ROS. The research indicates that physiological concentrations of butyrate and other SCFAs induce ROS, which transiently alters the intracellular redox balance. This process results in the inhibition of the NEDD8-conjugating enzyme Ubc12, leading to the loss of neddylated Cullin-1 and subsequent suppression of the NF*κ*B pathway. This suppression affects the ubiquitination and degradation of I*κ*B*α*, an inhibitor of NF*κ*B. These findings suggest the link between metabolic products of intestinal flora and key inflammatory and proliferative signalling pathways in the epithelium is a predisposed mechanism by which SCFAs modulate ROS production and influence inflammation [96].

Neutrophils activated by butyrate inhibit the ROS production via a PTX-sensitive way, while acetate shows the complete opposite outcome [58]. After purposefully inducing colitis using 2% Dextran Sodium Sulfate (DSS), Sina *et al.* [97] experimentally demonstrated that GPR43-deficient (GPR43-/-) mice showed a reduction in inflammatory mediator activities and in tissue damage. Results of Maslowski *et al.* [98] are contradictory to Sina *et al.* and similar to Masui *et al.* [91]. Their experiments showed poor inflammation resolution in GPR43-deficient mice. The former experiment implies a pro-inflammatory role of GPR43, while the latter implies an anti-inflammatory role. It can be concluded that GCPR activation via SCFAs can lead to pleiotropic effects. Also, Kim *et al.* [57] reported similar results to that of Sina *et al.* [97], but different means for colitis induction (ethanol, TNBS) were used. In addition, researchers showed that the inflammatory response in colitis was Th1- and Th17-cell mediated when colitis was induced by Trinitrobenzene Sulfonic Acid (TNBS), while DSS-induced colitis shifted from a Th1/17- to a Th2-mediated inflammatory response. These contrasting responses are indicative of varied roles of GPR43 depending on methods used for inflammation (colitis) induction [99]. Again, studies have not been conducted on humans, thus lacking in providing similar effects on humans [90].

Elevated levels of SCFAs can also have detrimental effects and be linked to inflammatory conditions. Nafday *et al.* [100] reported colonocyte injury in rats when exposed to extremely high doses of SCFAs, particularly butyrate at 300 mM and pH 4.0, compared to the normal range of 70–140 mM at pH 5.5 in the proximal region of colon. Histological examination showed that the severity of enterocolitis was age-dependent.

Recent studies by Shimizu *et al.* [101] reported the high faecal pH in patients having the systemic inflammatory response syndrome (known as SIRS). The alteration in pH, caused by the anionic nature of SCFAs, when combined with disrupted SCFA levels and the presence of pathogenic *Staphylococci* and *Pseudomonas* in patients with SIRS, suggests the presence of gut motility disorders, also known as intestinal dysmotility, which can lead to injury and inflammation of the gut lining [101,102]. This deteriorated gut lining, immune system jeopardy and altered SCFA-producing gut bacteria could cause a syndrome called multiple organ failure syndrome, or MODS [103,104], all paving a way towards gut-to-body axis in illness. Furthermore, a study by Desoignie and Sellin showed that propionate induced the intracellular acidification in epithelial cells of rabbits when supplemented with varied concentrations of propionate. Administering varied concentrations of SCFAs resulted in an abrupt episode of acidification of colonocytes [105], although this low pH (acidification) was found to be balanced shortly after administration of SCFAs. This implies that a sudden surge of propionate causes a shift in pH that could be harmful to intestinal cells.

**Fig. 2.**
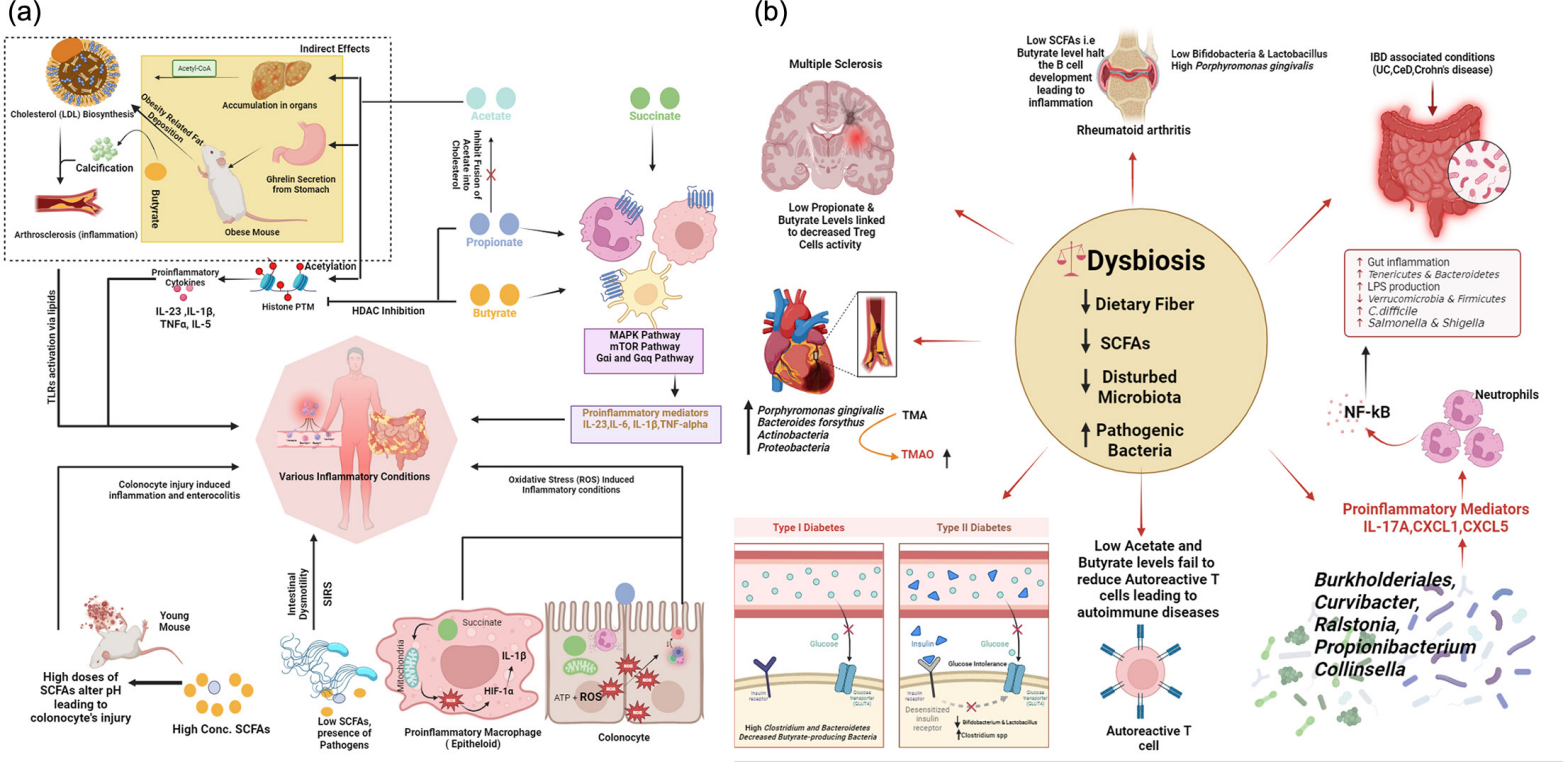
(**a**) Acetate is a precursor in pathways of lipogenesis and cholesterol synthesis. Cholesterol (LDL) deposited in vascular tissues of various organs causes blockage and triggers TLRs which in turn upregulate pro-inflammatory pathway genes. Butyrate enhances calcification which can block blood vessels. SCFAs in excessive amounts can produce oxidative stress response via ROS production, which can cause inflammation if the process is dysregulated. SCFAs can activate immune cells via GPRs with resultant release in pro-inflammatory cytokines through various signalling pathways. SCFAs on young mice impart pro-inflammatory situations and exacerbate colitis. (**b**) Dysbiosis of gut microbiota and SCFAs leads to various diseases.

Although SFAs are mostly described as protective therapeutic microbiota metabolites on cancerous cells and autistic brain cells, many studies have been conducted on various cell lines where SCFAs, especially butyrate, increase mitochondrial ROS production and oxidative stress milieu, leading to proliferative, apoptotic and inflammatory gene expression [106,110]. Presence of succinate actively promotes excessive ROS in non-cancerous neural cells causing injury in rats as reported by Zhang *et al.* [111]. Similarly, another study by Mills *et al.* [87] showed macrophages with high levels of succinate oxidation in mitochondria caused high ROS to stimulate naïve macrophages into pro-inflammatory macrophages (M1 type) (Fig. 2). ROS, especially superoxide, stimulates IL-6 and promotes epithelial dysfunction and fibrotic hypertrophy [110]. The above-mentioned results of Kumar *et al*. [92] show loss of neddylated Cullin-1 helps in the compensatory mechanism of inactivating ROS, but with extremely elevated levels of SCFAs, this adaptability could be lost, leading to injury-related processes and ultimately ending up being cytotoxic to the cell [92]. Excessive levels of SCFAs are deteriorating particularly for premature intestines, as high SCFA concentrations caused intramural haemorrhage in a neonate rabbit model reported in an investigation by Clark *et al.* [112].

A growing body of experimental work indicates that gut microbiota-derived metabolites, including SCFAs and metabolites such as succinate, can act as context-dependent pro-inflammatory triggers in extra-intestinal tissues. For instance, Papatriantafyllou showed that succinate accumulates in activated macrophages and stabilizes HIF-1*α* to drive IL-1*β* production, identifying succinate as a bona fide pro-inflammatory immunometabolite [113]. Succinate is associated with stabilization of HIF-1*α* and triggers IL-1*β*, noting that high succinate is seen in rheumatoid arthritis (RA), IBD, obesity and atherosclerosis [114,116]. Pluznick *et al.* [35] demonstrated that acetate and propionate activate the olfactory receptor Olfr78/human OR51E2 in juxtaglomerular cells and arterioles, increasing cAMP and renin secretion, a pathway that links SCFAs to vascular tone and inflammatory/pressure regulatory responses in the kidney and vasculature. Acetate can drive a brain-*β*-cell axis (via parasympathetic activation) that increases insulin release, hyperphagia and downstream metabolic inflammation in rodent models, indicating an acetate-driven route to systemic pro-inflammatory metabolic sequelae, including obesity [117,118]. Recent human cell and animal studies have described NLRP3 inflammasome activation by butyrate and propionate in macrophages under certain conditions, demonstrating direct pro-inflammatory innate immune activation by SCFAs (NLRP3/IL-1*β*) [119]. Several reports also emphasize concentration and context dependence: low concentrations of butyrate are anti-inflammatory for many cell types, but higher concentrations or particular metabolic states can shift butyrate’s action towards pro-inflammatory outcomes in macrophages and microglia [120]. A recent dose-response study showed that low butyrate suppressed LPS-driven cytokine production, while high butyrate concentrations potentiated pro-inflammatory responses in human macrophages. Targeted organ studies show SCFA-related pro-inflammatory contributions in extra-intestinal settings explain SCFA dysregulation correlates with macrophage M1 polarization and inflammatory signalling in liver (NASH/HSOS models), and butyrate/propionate can function as danger signals activating innate immune pathways in systemic macrophages and lung immune cells under disease-relevant conditions, demonstrating that SCFAs contribute to inflammatory phenotypes beyond the gut [121,122]. Rutting *et al.* reported that high concentrations of SCFAs synergized with TNF-*α* to elevate IL-6 and IL-8 release from human lung fibroblasts and airway smooth muscle cells via an FFAR3–p38-MAPK mechanism [123]. Together, these recent studies demonstrate that SCFA-derived signals can act as pro-inflammatory mediators in non-intestinal organs as well.

#### Epigenetic regulation: SCFA-mediated histone acetylation and inflammation

Histone deacetylation catalysed by acetylase enzymes gains central importance in transcription and translation of various genes involved in pro-inflammatory cytokine production, which is responsible for many inflammatory and metabolic diseases. SCFAs function as inhibitors of histone deacetylation enzymes and halt the process of deacetylation that suppresses the production of inflammatory mediators [124]. Reports showed successful inhibitions of HDACs by butyrate and propionate with varying degrees, but no effect by lactate was observed in cancerous cells [125]. Similarly, acetate but not butyrate and propionate has been known for not being an inhibitor of HDAC in endothelial cells [126]. This effect is speculated to be tissue-dependent, where it causes inhibition of deacetylation in some tissues while promoting the deacetylation process in others [127]. Contrary results are also present in the literature, where butyrate treatment on cancer cell lines decreased acetylation and subsequently increased deacetylation of genes [128]. Although acetate is studied to be involved in acetylation of histone [129,131], this role of acetate in increasing acetylation is suggestive of regulation of many genes which are involved in pro-inflammatory cytokine production. The experimental report of Berndt *et al.* [132] demonstrated that DCs activated by Lipopolysaccharide (LPS)-induced colitis and treated with butyrate showed significant outcomes: it suppressed anti-inflammatory cytokine IL-12 and upregulated pro-inflammatory IL-23 by HDAC inhibition pathway. The study further strengthened the stance on the pro-inflammatory role of orally administered butyrate, which exacerbated the DSS-induced colitis conditions in rats. Similarly, other studies show effects of butyrate, acetate and propionate on different cell lines through acetylation and inhibition pathways of deacetylation, resulting in production of various pro-inflammatory mediators such as IL-1*β*, TNF-*α*, NF*κ*B and IL-5 [133,135].

#### TLR-mediated pro-inflammatory pathways

Extensive study done by Lin *et al.* [134] shows that in response to several TLR ligands and TNF-*α*, SCFAs, namely butyrate (C4) and propionate (C3), augment NF*κ*B activation. This improvement is similar to the effects of the TSA-mediated HDAC inhibition and is connected to modifications in histone acetylation. Pro-inflammatory and chemotactic cytokine responses were selectively modulated in colonic epithelial (HT-29 cells) in response to flagellin; butyrate and propionate boosted TNF-*α* transcription while lowering IL-8 and MCP-1 levels. According to these results, SCFAs may be able to regulate the local inflammatory response by increasing pro-inflammatory signals (such as TNF-*α*) and reducing the expression of chemokines (IL-8 and MCP-1), which inhibit the recruitment of immune cells [134]. There is a correlation between butyrate concentration and TLR5 expression in the colon, indicating TLRs’ involvement in inflammatory responses. Expression of TLRs decreases with decrease in butyrate concentration from proximal end to distal end of colon [136]. Due to the complexity in nature, time and cell-type-specific effects of SCFAs, a general conclusion on their impact on cytokine responses is a bit challenging. Nonetheless, SCFAs’ promotion of local TLR responses, which helps induce inflammation, is not worth ignoring the possibility of being a factor in inflammatory diseases in large and complex multifactorial arenas of combat between health and disease. Multifaceted effects and biphasic responses of SCFAs in various cell types and hosts are presented in Table 1.

**Table 1.** Pleiotropic effects of SCFAs on various cells

Various SCFAs	Study model	Mechanism/parameters studied	Outcome	References
Acetate	Rat liver slices	Acetyl-CoA pools' intracellular dilution and compartmentalization during cholesterogenesis	For the production of cholesterol, acetate supplies a cytosolic pool that is not in isotope equilibrium with the mitochondrial pool	[64]
Acetate	Conscious mice	Infusion of stable isotopes in the caecum, metabolism by host	Acetate and butyrate are used for palmitate and cholesterol synthesis; propionate is used for glucose production	[67]
Acetate	Obese mice on a high-fat diet	Acetate administration, mRNA expression levels of adipogenicity and lipid metabolism genes, signalling pathways	Increased body weight, food intake, lipid levels, anti-inflammatory factors and adipocyte differentiation	[320]
Acetate	TSOD mice	Gut dysbiosis, plasma SCFA levels	Decreased plasma SCFA levels, increased propionate and butyrate, disrupted gut microbiota composition	[321]
Various SCFAs	Obese and normal-weight children	Gut microbiota composition, SCFA levels in stool	Altered gut microbiota in obese children, higher SCFA levels and elevated fermentation activity	[322]
Various SCFAs	Obese and normal-weight children	Dominant gut microbiota genera, stool SCFA and BCFA content	Lower SCFA concentration in obese children’s stool, predominance of *Firmicutes*	[323]
Receptors mediated dichotomous responses of SCFAs				
Mixture of SCFAs	High-fat diet-fed mice	FFAR2/3 expression, body weight, cholesterol, inflammatory mediators	Decreased MCP-1, total cholesterol, IL-1*β*, IL-6 and body weight; elevated adiponectin and fat oxidation	[324]
Individual SCFAs	High-fat diet-fed mice	Free fatty acids, glucose, inflammatory indicators and plasma cholesterol	Decreased weight gain, visceral fat mass, IL-1*β*, IL-6; enhanced sensitivity to insulin and oxidation of fat	[325]
Butyrate, propionate	FFAR2-KO mice	Body fat mass, RER, energy expenditure and FFAR2 expression	Reduced RER, higher energy expenditure, higher core body temperature and decreased body fat mass	[326]
Butyrate, propionate	FFAR2/3-KO mice	Gut hormonal release, glucose tolerance	Impaired gut hormone secretion (PYY, GLP-1), disrupted glucose tolerance	[327]
Histone acetylation/deacetylation pleiotropic effects of SCFAs				
Acetate	Rat model	Histone acetylation, HDAC inhibition	Reduced neuroinflammation, IL-1*β* levels	[328]
Acetate	Mouse macrophages	Glycolysis, increased histone acetylation (H3K9)	Enhanced IL-1*β* production, suggesting potential pro-inflammatory activity	[329]
Acetate	Human macrophage cell line	Acetyl-CoA synthetases, histone acetylation	Increased pro-inflammatory mediators IL-6, TNF-*α*	[133]
Butyrate	Human intestinal epithelial cells	HDAC inhibition, GPR109A activation	Increased CCL20 secretion imparts dual pro and anti-inflammatory effects	[330]
Butyrate, propionate	HEK293, HeLa cells	TLR–NF*κ*B signalling, histone acetylation	Altered cytokine/chemokine expression, NF*κ*B-induced inflammation, damped Il-8	[134]
Butyrate	Jurkat cells	HDAC inhibition, hyperacetylation (H3, H4)	Increased IL-5 production leading to promote allergic inflammation	[135]
Butyrate	Asthmatic BALB/c mice	HDAC inhibition, NF*κ*B suppression	Reduced asthma-induced inflammation severity	[331]
Acetate	Rat brain and liver	HDAC inhibition, acetyl-CoA increase	Increased histone acetylation, reduced HDAC activity and suppression of inflammatory milieu.	[131]
Contrasting results on colitis				
Acetate, butyrate, propionate	Newborn Sprague-Dawley rats, Neonate rabbits	Luminal administration, mucosal injury scoring Promotion of intramural haemorrhage	Greater mucosal injury in younger rats, reduced injury in mature rats Neonate rabbits showed enterocolitis.	[100,112]
Acetate, butyrate, propionate	Male BALB/c mice	Cancer linked to colitis brought on by AOM/DSS and SCFAs in drinking water	Enhanced inflammation, decreased pro-inflammatory cytokines IL-6, TNF-*α* and IL-17 and decreased tumour incidence and size	[332]
Oxidative stress response and dual SCFA outcomes				
Butyrate	Human intestinal epithelial cells	Inhibition of NF*κ*B pathway via ROS and neddylation	Inhibition of NFκB pathway, suppression of inflammation	[92]
Butyrate	Hepatic cells	miR-22-mediated ROS production and apoptosis	Induction of apoptosis, inhibition of cell proliferation	[107]
Butyrate	LT97 and HT29 colon cancer cells	Chemo preventive effects modulated by ROS	Growth inhibition, apoptosis, modulation of cell cycle proteins	[108]
Acetate, propionate, butyrate	Colorectal adenocarcinoma cells	ROS production, metabolic and transcriptomic changes	Elevated ROS, modulation of metabolic and transcriptomic antitumour signatures	[109]
Propionate	Murine macrophage cell line, J774-A1 and rat model	Anti-inflammatory and antioxidant responses	Decreased expression of pro-inflammatory mediators (COX-2, iNOS), enhanced antioxidant enzymes	[333]
Propionic acid	Rat model of ASD	Lipid metabolism alterations, locomotor behaviour	Increased locomotor activity, altered lipid profiles and neuroinflammation due to propionic acid infusion in brain	[334]
Propionic acid	Human neutrophils	IP3 formation, Ca2+ mobilization	Superoxide generation in presence of Ca2+ionophore, contrary to antioxidant role of SCFA	[335]
SCFAs biphasic influence on immune cells				
Succinate	Mouse macrophages	LPS stimulation leading to glycolysis and ROS production	Promotes pro-inflammatory state via ROS production	[87]
Succinate	Mouse macrophages	GPR91 receptor activation, autocrine/paracrine signalling	Enhances IL-1*β* production, contributes to arthritis inflammation	[305]
Succinate	Mouse macrophages	SUCNR1-independent suppression of IL-6, TNF, NO	Anti-inflammatory effects, context-dependent response	[336]
SCFAs (General)	Human monocytes, PBMC	GPR43-mediated PGE2, cytokine and chemokine regulation	Distinct anti-inflammatory activities, inhibition of TNF-*α*, IFN-*γ*	[337]
Butyrate, propionate	Human DCs, CD8+ T cells	Inhibition of IL-12, IL-23 secretion	Reduces CD8+T cell activation, potential in anti-cancer therapy	[338]
Butyrate, propionate	Mouse and human B cells	Inhibition of histone deacetylation, miRNA regulation	Impairs antibody responses, inhibits autoimmunity	[339]
Butyrate	Germ-free mice, CD8+ T cells	Promotion of oxidative metabolism, metabolic rewiring	Enhances CD8+T cell memory potential, improves recall responses	[340]
Butyrate, phenylbutyrate, phenylacetate	RAW 264.7 cells	Repression of NFκB, ERK1/2 phosphorylation	Anti-inflammatory effects, reduced IL-6, TNF-*α*, enhanced IL-10	[341]

Inflammation, which inherently is a protective mechanism, can eventually lead to chronic form. Chronic inflammation is a causative villain in cancer, cardiovascular disease and neurological problems. Ageing, recurrent infections, chronic illnesses, obesity and environmental pollutants can contribute to this notorious shift [137,138]. Oxidative stress and free radical generation worsen the inflammatory condition [139]. SCFAs may paradoxically contribute to chronic inflammation when their pro-inflammatory role surpasses their anti-inflammatory capabilities, particularly if this role becomes dysfunctional or dysregulated, leading to the overstimulation of immune cells and receptors, ultimately leading to chronic inflammation. Understanding the causes of chronic inflammation is critical for finding ways to reduce its negative health effects. More research studies on SCFAs are necessary because their role in perpetuating inflammation highlights the complexity of immune responses and the need for balanced therapies to prevent chronic inflammatory disorders.

#### SCFAs associated dysbiosis and inflammation

The gut microbiota, a microbial symbiont, is present in a harmonious relationship with the host gut, making significant contributions not just to the gut but also to the overall physiology of the host [79]. Dysbiosis is the imbalance or alteration in normal distribution of microbiome accompanied by proliferation of pathogenic bacteria, which comes with its own set of problems, with their unique structural and metabolic footprints leading to the notorious effects on the body of the host, including various inflammatory processes and conditions [79]. It is now accepted that dysbiosis affects the production of SCFAs and impairs GPCR signalling, thereby contributing to various diseases [140]. These structural and metabolic chemicals, such as LPS, peptidoglycan, lipoic acid and flagellin, nucleic acids and toxins, are collectively known as pathogen-associated molecular patterns (PAMPs) that cause inflammation. These PAMPs stimulate immune cells of innate and adaptive immunity and differentiate these cells into inflammatory kinds via interaction with specialized structures known as PRRs (TLRs, NLR and scavenger receptors) [141]. Inflammation caused by these processes is the main culprit in disruption of normal functions, rendering idiopathic conditions like IBD, RA, ulcerative colitis (UC), diabetes, etc., and real perpetrator of fibrotic conditions [142] (Fig. 2).

Samanta and colleagues [143] reported that UC-inflicted mice showed altered gut microbiota compared to healthy mice. Metagenomic analysis of faeces samples revealed a high concentration of species from the Verrucomicrobia phylum and a low concentration of the Tenericutes phylum. Infections by *Enterobacteriaceae* species such as *Salmonella* and *Shigella* can lead to neutrophil circulation, reducing SCFAs, which are otherwise crucial for gut health. This inhibited production of SCFAs can be correlated with pathogen growth, leading to inflammation and tissue damage in the intestinal mucosa [144]. Experimental studies have demonstrated that dysbiosis increases susceptibility to enteric infections, and perturbations in the native gut microbiota induced by antibiotics can cause inflammation. Studies have linked the presence of *Escherichia coli* in DSS-induced colitis animals, elevated *Clostridioides difficile* levels in hospitalized patients due to antibiotics and similar pathogen increases in murine models, highlighting the correlation between enhanced systemic pathogen circulation and inflammasome activity [145].

Intestinal epithelial cells are activated by SCFAs. This causes the expression and release of cytokines like IL-1, IL-6, IL-12 and IL-18, which are important for immune responses. In these cells, GPR43 also facilitates the activation of inflammasomes [57,146]. Furthermore, GPR43 acts as a chemotactic receptor for neutrophils, which may guide these leucocytes to regions rich in SCFA, such as the gut lumen and lamina propria. The specific functions and cell types involved remain unclear; however, it is known that GPR41 and GPR43 are extensively expressed in the pancreas [147,148], where decreased levels were hypothesized to be associated with type 1 diabetes (T1D). The study conducted by Mariño *et al.* [149] discovered an inverse relationship between the microbial metabolites, such as butyrate and acetate, and critical disease characteristics in non-obese diabetic mice. Using tailored diets, the researchers discovered that even after immunotolerance breakdown, each diet offered strong protection against diabetes. Metabolites of acetate and butyrate reduced the frequency of autoreactive T cells, improved the function of regulatory T cells and strengthened intestinal integrity. This indicates a natural strategy to combat autoimmune illnesses that are dependent on T cells. Type 2 diabetes is also linked to reduced levels of SCFAs and SCFA-producing gut microbes, as reviewed and discussed in [150,153]. In T1D, gut microbiota plays a crucial protective role with therapeutic effects. Increased *Clostridium* and *Bacteroides* and decreased lactate- and butyrate-producing species have been correlated with T1D [154]. Patients with T2D often have been found to present an excess of *Clostridium* and a reduction in *Bifidobacterium* and *Lactobacillus* spp. And this microbiota alteration is hypothesized to be causing glucose intolerance and metabolic complications [155]. Supplementing with *Bifidobacterium* can improve glucose tolerance and can reduce insulin resistance [156,157].

Dysbiosis of gut microbiota, caused by changes favouring pathogenic bacteria such as *Prevotella intermedia*, *Porphyromonas gingivalis* and *Bacteroides forsythus*, leads to atherosclerotic cardiovascular disease (ACVD), as demonstrated in a study by Haraszthy *et al.* [158]. Similar studies by Ziganshina *et al.*, Voronina *et al.* and Karlsson *et al.* revealed altered and elevated species of gut microbes belonging to the order *Burkholderiales*, genera *Curvibacter*, *Ralstonia*, *Propionibacterium* and *Collinsella*. Particularly, bacteria like *Collinsella* produce pro-inflammatory cytokines and chemokines such as IL-17A, CXCL1 and CXCL5. These molecules stimulate neutrophil recruitment and NF*κ*B activation [159,162]. Dysbiosis also impacts (reduces) the levels of SCFAs, particularly butyrate, which typically regulate inflammation and stabilize plaques, thereby worsening atherosclerosis [163]. Additionally, dysbiosis leads to increased synthesis of trimethylamine (TMA) from dietary choline produced by opportunistic bacterial species like *Actinobacteria* and *Proteobacteria*. TMA is then converted to trimethylamine-*N*-oxide (TMAO) in the liver through FMO3. Elevated TMAO level is linked to a higher cardiovascular risk, disrupted cholesterol metabolism and increased inflammation, underscoring the complex interplay between gut microbiota and metabolites and the development of ACVD [164,165].

Emerging studies show the SCFAs’ correlation with allergic diseases of various types. Gut microbiota dysbiosis and disrupted levels of SCFAs are hypothesized to link with impaired and dysfunctional immunity, promoting various hypersensitivity reactions and allergies [166]. Allergies are the prime cause of inflammation [167] and hence are speculated to be associated with inflammatory diseases, including IBD and gut-related ailments [142,168,171].

Coeliac disease (CeD) is characterized by inflamed small intestine due to villous atrophy, which can cause malnourishment, cancer and other gastrointestinal issues. Gut microbiome dysbiosis and low SCFAs, such as butyrate and acetate production, have an impact on CeD [172,173]. Gut microbiota dysbiosis lowers SCFA levels, especially butyrate and propionate, which are linked to decreased Treg cell activity and increased inflammation. There is an inverse relationship between pro-inflammatory cytokine levels and SCFA levels, implying a balance-driven link with multiple sclerosis (MS) disease [174,178]. Systemic lupus erythematosus (SLE) and RA are influenced by altered gut microbiota compositions and SCFA deficit availability. SLE patients have fewer SCFA-producing *Firmicutes* and more pro-inflammatory mediator-producing Bacteroidetes, which leads to Th17 differentiation and causes inflammation [178,181]. RA patients had lower SCFA levels, which are necessary for regulatory B cell development and gut–joint axis integrity. Butyrate deficit in RA condition enhances inflammation and intestinal permeability [182,183].

## Role in salutogenesis

SCFAs are either recognized by GPCRs as effectors for leucocytes in downstream signalling cascade processes or are absorbed through transport protein channels such as MCT1 and SMCT1, where they are utilized in cell metabolism and regulate various inflammatory conditions. Gut and other organs associated with inflammatory processes are regulated when SCFAs act on leucocytes present and endothelial linings via these receptors and channels [184]. Macrophages are the major WBCs, which serve as trigger points for inflammation in conditions such as dysbiosis and cell-dependent effects, thus releasing pro-inflammatory mediators, namely TNF-*α*, IL-1*β*, nitric oxide (NO) and IL-6, as described somewhere else in this article. In their regulatory role, SCFAs are associated with suppression of these pro-inflammatory chemicals as well as enhanced production of anti-inflammatory cytokines and mediators [22].

HDACs, enzymes that induce transcriptional repression of regulatory genes by removing acetyl from histone proteins, are inhibited by SCFAs. Through this inhibition, SCFAs can stimulate neutrophils and mononuclear blood cells, reducing the levels of pro-inflammatory chemicals such as TNF, which subsequently suppresses NF*κ*B [185].

### Innate immune cells

Macrophages can play both hero and villain roles in inflammation, either differentiating into anti-inflammatory or pro-inflammatory macrophages, respectively. A conditional dramatic shift turns naïve macrophages either into pro-inflammatory (M1) macrophages with the ability to induce an inflammatory response by expressing pro-inflammatory cytokines and recruiting immune cells, or anti-inflammatory (M2) macrophages with the ability to fully resolve inflammation by enhancing the expression of anti-inflammatory mediators [186]. This phenotypic shift in role is attributed to various cellular conditions such as metabolite dysbiosis, metabolic reprogramming and stress situations, as well as SCFA stimulation [58,187]. The pro-inflammatory role has been discussed elsewhere in this review. Macrophages promote anti-inflammatory activity throughout the body’s organs, including the gut [188]. The most studied mechanism of macrophages to produce anti-inflammatory mediators is through the inhibition of HDACs upon activation by SCFAs, especially butyrate, since butyrate shows high potency to act/stimulate macrophages *in vivo* and *in vitro*. SCFAs activated macrophages obtained from intestine in study by Chang *et al.* [189] and from peripheral blood in study by Usami *et al.* [190] via HDAC inhibition and led to the reduced expression of IL-6, IL-12 and TNF-*α*. Another study by Park *et al.* [191] reported that nitric oxide (NO), a crucial virulence factor in *Staphylococcus aureus*-induced inflammation, was suppressed by SCFAs such as butyrate and propionate in mouse macrophages. Additionally, the expression of NF*κ*B, IFN-*β*, STAT1 and inducible NO synthase (iNOS), which are necessary for iNOS transcription, was reduced by these SCFAs. Moreover, HDAC inhibitors reduced NO synthesis in macrophages, indicating that SCFAs might be able to lessen *S. aureus* inflammatory responses [191]. mTOR regulates NF*κ*B expression in macrophages by targeting the inhibitor component I*κ*B*α*, which in turn causes inflammation [192]. Bacterial components encourage enhanced mTOR activity, resulting in elevated pro-inflammatory cytokines (such as TNF-*α* and IL-6) and decreased IL-10 production. It has been demonstrated that reduced mTOR activity by butyrate in macrophages leads to a decrease in expression of pro-inflammatory cytokines as well as an increase in IL-10 expression, with the effects being mediated via HDAC3 inhibition [193].

SCFAs produced by the populated gut microbiota protect against inflammation and potential injury in the gut and throughout the body by regulating tight epithelial junctions in the gut and secreting mucus to create a protective barrier. These SCFAs also stimulate the production of antimicrobial peptides and chemicals, including REGIII*γ*, *α*,*β*-defensins and cathelicidin LL-37, that defend against potential pathogens and associated inflammatory conditions [25,194, 195]. Butyrate plays a crucial role in transforming gut macrophages into an anti-inflammatory type, as described by Schulthess *et al.*, as gut-derived butyrate-supplemented macrophages in a murine model showed M2 properties [196]. Additionally, a study trial on ten obese and nine healthy human subjects showed that supplementation with butyrate (~4 g daily over 1 month) decreased Pam3CSK4-induced TNF-*α* and LPS-induced IL-6 in oxLDL-trained immunity (in peripheral blood mononuclear cells) not only in healthy but also in obese individuals with metabolic syndrome [197].

SCFAs play a crucial role in regulating neutrophils by reducing pro-inflammatory cytokines and promoting the resolution of inflammation. Neutrophils, the most prevalent innate immune cell in bone marrow and peripheral circulation, are vital for inflammation control [198]. Through the GPR43 receptor, SCFAs facilitate chemotactic recruitment to inflammatory sites and increase l-selectin expression, influencing neutrophil behaviour. Intracellular pathways like P38 protein kinase and G proteins Gi/o and Gq are activated in this process. As mentioned earlier, SCFAs such as C4 inhibit neutrophils from producing pro-inflammatory cytokines (TNF-*α*, IFN-*γ* and IL-6) and chemokines (IL-8, CXCL1, CCL3, CCL4) that are implicated in diseases like pancreatitis and colitis [52,53, 199, 200]. These actions underscore the role of neutrophils in dampening inflammatory responses, mediated through HDAC-dependent pathways. Butyrate inhibits the growth of pathogenic bacteria and pathogen-induced production of pro-inflammatory cytokines in neutrophils by directly inhibiting HDAC3 and 9, suppressing NF*κ*B and enhancing PPAR-*γ* activity [201]. Studies on rat models have shown that butyrate reduces the production of pro-inflammatory cytokines like chemoattractant-2 and TNF-*α* by neutrophils, both *in vivo* and *in vitro* [188,202]. However, in neutrophil cells, acetate has been found to promote inflammation by activating inflammasomes [88].

Propionate and butyrate have been reported to reduce l-selectin expression on the surface of neutrophils [97]. Nanoparticles of lipid 3*β*-Butanoyloxycholest-5-ene (Cholesteryl Butyrate) have been shown to express an anti-adhesive effect on neutrophils in the endothelial lining [203]. This suggests that SCFAs have an impact on neutrophil recruitment to the site of inflammation and in this way may help lessen it. However, contradictory results regarding the effects of SCFAs on l-selectin and other adhesive molecules are also present in the literature. While without altering *β*2 integrin mRNA, SCFAs exacerbate inflammation, trigger neutrophil migration to inflammatory areas and increase l-selectin expression on neutrophil granulocytes [59,204]. Based on these results, it can be speculated that SCFAs influence the interaction between neutrophils and endothelial cells due to their impact on endothelial cells rather than on neutrophils [53].

Among other innate immune cells, innate lymphoid cells (ILCs), basophils, eosinophils and natural killer (NK) cells are all important in the process of reducing inflammation. The metabolic alterations brought about by IL-12- and IL-15-induced NK cell activation are essential for increased receptor expression and cytokine production. SCFAs such as C4 promote an anti-inflammatory milieu by suppressing pro-inflammatory cytokine production (TNF-*α*, IFN-*γ*, IL-22) and surface receptor expression (TRAIL, NKp44, NKp30, NKG2D) in NK cells activated by IL-12/IL-15. This inhibition highlights the function of NK cells in immunological regulation and inflammation resolution by affecting regulators like mTORC1 and c-Myc and involving pathways like HDAC modulation [183,205,207]. When eosinophils are activated in response to an allergen, they release pro-inflammatory substances. However, SCFAs C3 and C4 control this process by inducing apoptosis and preventing migration and the production of cytokines, thus functioning as a compensatory mediator in many inflammatory conditions, including allergies and eosinophil-associated diseases [22,208,211]. Less research has been done on SCFA interactions with basophils, although they exhibit higher activation with C2 treatment and improved IL-3-induced responses with C3 and C4, such as degranulation and suppression of cytokine production [22,212]. Similarly, through GPR receptors and HDAC pathways, SCFAs modulate ILCs, especially ILC3s, which are essential for IL-22 synthesis and barrier integrity. This influences cytokine secretion and inflammation regulation. The various functions of SCFAs in immune cell modulation and inflammation regulation have been explored here [22,88, 213,215].

SCFAs have a significant impact on the regulation of adaptive immune cells, particularly T cells and B cells. They also play an essential role in suppressing and mitigating inflammatory processes generated by lymphocytes.

#### T cells

The production of SCFAs by certain strains of the gut microbiota triggers the transforming growth factor-beta (TGF-*β*) response, facilitating the development and expansion of regulatory T lymphocytes (Tregs) in the colon, as shown in studies on mice by Smith *et al.* [216]. These cells are essential for ensuring intestinal homeostasis. Post-translational modification of T cells leads to the formation of Tregs that express the FoxP3^+^ protein on their surface [217]. Tregs play a crucial role in producing IL-10 and other anti-inflammatory cytokines, which help in reducing intestinal inflammation [218]. SCFAs like butyrate (C4) promote Treg differentiation by enhancing histone H3 acetylation at the FoxP3+ promoter through epigenetic changes. Additionally, by stimulating the production of IL-10 and enhancing the anti-inflammatory response through interaction with macrophages and DCs via the GPR109A receptor, SCFAs indirectly promote Treg proliferation [219,221].

It’s interesting to note that while pentanoate (C5) increases IL-10 production and glycolysis through the mTOR activation route, it does not directly increase Treg numbers. In contrast, butyrate enhances Treg proliferation [222]. Additionally, SCFAs regulate T lymphocyte metabolism by inhibiting HDAC, a process that promotes the differentiation of naive T cells into effector T cells such as Th1 and Th17, which are involved in inflammatory responses. By facilitating rapid effector function through GPR-dependent pathways and HDAC inhibition, SCFAs enhance CD8+ T cell metabolism during antiviral responses. This could be a potential strategy in cancer therapy and cancer-related inflammatory conditions [220,223,227].

#### B cells

For B cells to proliferate, differentiate and produce antibodies, they need fatty acid synthesis, glycolysis and oxidative phosphorylation [228]. SCFAs influence B cells to differentiate into plasma cells and boost the generation of IgA and IgG antibodies, as reported by Ishikawa and Nanjo [229] in a study on probiotic-fed mice. This led to elevated serum IgA and IgG levels, as well as increased SCFA content and IgA levels in the large intestine and faeces. This indicates that prebiotics, which enhance the production of SCFAs, may support B cell development and antibody formation [22]. Acetyl-CoA, a precursor essential for fatty acid synthesis and ATP generation in the TCA cycle, is produced during the metabolism of SCFAs and is critical for the energy metabolism and function of B cells. Acetyl-CoA involves acetylation of histones, which in turn is helpful in the HDAC inhibition pathway in B lymphocytes [22,230, 231]. GPR receptor mediation and HDAC inhibition enable SCFAs to proliferate B cells by altering gene expression, enhancing T helper cell glucose absorption and activating Tfh for B cell maturation and antibody production, while also increasing regulatory B cell development and cytokine production, all contributing to the regulation of inflammation in one way or the other [232,233].

### SCFA and other inflammatory conditions (gut–body link)

IBD is a chronic intestinal disorder that includes CD and UC. While UC is restricted to the colon and causes ulcerations, profuse bleeding, toxic megacolon and fulminant colitis inside the colon, CD is typified by a transmural inflammatory condition affecting the entire digestive tract [234]. A complex interaction of genetic, environmental, microbial, epithelial and immunological variables is involved in the pathophysiology of IBD. Through a variety of pathways involving diverse receptors and immune cells, SCFAs, such as acetate, propionate and butyrate, play crucial roles in regulating IBD. Higher SCFA levels are obtained via increased dietary fibre consumption, and these levels are essential for preserving the colon’s immunological barrier function and promoting the production of anti-inflammatory agents [235].

Acetate interacts with the GPR43 receptor, mediating anti-inflammatory effects by inhibiting HDAC activity [53]. Propionate activates GPR43 and inhibits HDAC, promoting Treg proliferation and function [22]. Butyrate’s influence is extensive, engaging both GPR43 and GPR109A receptors, inhibiting HDAC activity, suppressing inflammation in colonocytes and activating the NLRP3 inflammasome, which promotes IL-18 expression [184,236]. Its ability to induce Foxp3+ Treg cells and T cells that produce IL-10 strengthens its anti-inflammatory properties [237].

Pro-inflammatory cytokines like TNF-*α* influence the intestinal inflammation during IBD and exacerbate it to chronic form. This chronic inflammation impairs the butyrate absorption and subsequent metabolism. Inflammatory conditions suppress the upregulation of genes involved in the metabolism of SCFAs such as butyrate in patients suffering from CD and UC [238]. Dysbiosis is characterized by an imbalance in SCFA-producing bacteria, as seen mostly in IBD patients, emphasizing the importance of maintaining a healthy microbiota for SCFA production and intestinal health [239].

#### SCFAs and body organs

SCFAs are essential for immune system control and gastrointestinal homeostasis. By strengthening the intestinal barrier, they prevent diseases like MS, allergic asthma, cystic fibrosis (CF) and non-alcoholic fatty liver disease (NAFLD). In addition to regulating host immune responses and affecting intestinal epithelial barrier function, SCFAs facilitate metabolic interaction along the gut–lung, gut–liver and gut–brain axes and mediate immunological tolerance [226].

SCFAs in the liver assist in reducing disease-associated inflammation and mediate organ-specific processes, demonstrating their therapeutic promise in the fight against inflammatory disorders and infections. Their capacity to coordinate interaction between the colon and the liver causes favourable metabolic and metagenomic alterations, which benefit both the adaptive and innate immune systems [23]. SCFAs control bile acid production and its transportation; hence, they regulate NAFLD development [240]. SCFAs serve as a building block to monounsaturated long-chain fatty acid (MUFA) synthesis. These MUFAs regulate liver fibrosis and carcinogenesis via Wnt/beta/catenin signalling mechanism and stearoyl-CoA desaturase enzyme activity [241,244].

SCFAs help maintain lung health by regulating the gut–lung axis. This connection helps to avoid disorders like allergic asthma and improves general immune function in the respiratory system. SCFAs contribute to respiratory health by affecting the intestinal barrier and immunological responses [226]. An increasing number of people throughout the world are dealing with allergies. Dysbiosis of the gut microbiome has been associated with an increased risk of allergic diseases, including food allergies, allergic rhinitis, atopic dermatitis and asthma [166]. SCFAs have an important role in immune response regulation and are important in maintaining gut homeostasis [22]. Allergic asthma aetiology includes eosinophil infiltration into lung tissue, which is triggered by ILC2 and Th2 cells that produce cytokines IL-5, IL-13 and GM-CSF. Studies on SCFAs, notably acetate, propionate and butyrate [209], have demonstrated that SCFAs protect against allergic airway inflammation [98]. SCFAs inhibit eosinophil adhesion and migration, regulate eosinophil epigenetics via HDAC and alter immune cells such as mast cells, Treg cells, Th9 cells and DCs [245,246]. Recent research suggests that SCFAs decrease airway inflammation and eosinophil infiltration through GPR41, HDAC inhibition and Foxp3 promoter acetylation [247]. Furthermore, SCFAs boost immune-regulatory mechanisms by increasing Polymorphonuclear myeloid-derived suppressor cells (PMN-MDSCs) and boosting Treg cells, which reduce allergic airway inflammation. The therapeutic potential of SCFAs in airway inflammatory diseases requires more exploration [248].

SCFAs can protect the heart by regulating regulatory T cells (Tregs) and reducing inflammation. They decrease heart hypertrophy, fibrosis, vascular dysfunction and hypertension [249]. Propionate, in particular, is important for heart health since its absence in animal subjects during experiments shows Treg cell depletion and devoids the cardioprotective benefits [250]. SCFAs also protect cardiovascular disease development by increasing Treg cell expression and decreasing pro-inflammatory cytokines [249]. The inhibitory effects of these SCFAs on HDACs and NF*κ*B activity promote heart health [202].

SCFAs, including butyrate and acetate, can improve kidney health [249]. They minimize renal fibrosis and enhance kidney function. Butyrate therapy reduces the loss of intestinal tight junction proteins and colonic mucin vis-à-vis kidney protection [251]. Acetate dramatically lowers kidney fibrosis in hypertensive mice and imparts its protective properties [252]. SCFAs in the kidneys control GPCRs and immune function, boosting the intestinal immune network while inhibiting damaging metabolisms. This control helps to the overall preservation of renal function [97].

**Fig. 3.**
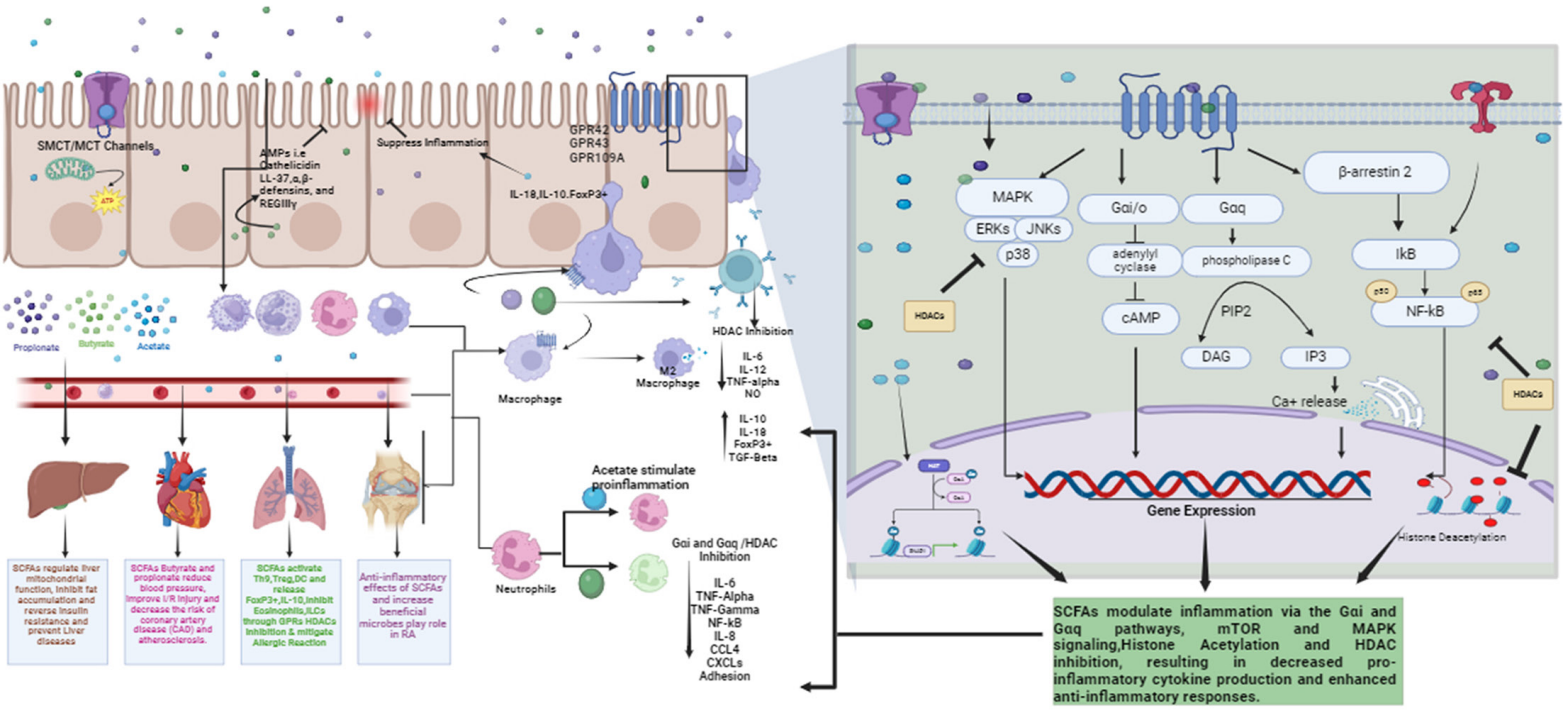
Systemic overview of SCFAs’ influence on different immune cells in colonocytes as well as in various organs, resulting in production of different anti-inflammatory cytokines and inhibition or reduction of pro-inflammatory cytokines through MAPK, G*α*i/G*α*q and HDAC inhibition. This leads to the immune system’s protective and homeostatic conditions in the body.

SCFAs also have an important role in the central nervous system (CNS), influencing metabolic and immunological interactions along the gut–brain axis. They aid in the regulation of inflammation and homeostasis inside the brain, possibly alleviating neurological diseases such as MS [253]. A thorough description of the positive effects of SCFAs on the brain and other organs is beyond the scope of this article. A holistic understanding of anti-inflammatory (modulatory) impact of SCFAs is illustrated in Fig. 3.

## Fibrosis: an exacerbated inflammation

The abnormal development of extra fibrous connective tissue in an organ or tissue during a reactive or reconstructive phase is known as fibrosis. It can impact the GI tract, liver, kidney, heart, skin and bone marrow, among other organs. Fibrosis arises from a combination of chronic inflammation and chronic trauma that causes an abnormal healing reaction [254]. Numerous factors can lead to fibrosis, such as persistent ischaemia, high blood pressure, frequent viral infections like hepatitis, environmental exposures like asthma, alcohol, diet, smoking, familial conditions like CF and alpha-1-antitrypsin deficiency and dysbiosis of gut metabolites, especially SCFAs [255]. The disease involves persistent damage to the parenchyma and dysregulation of repair, which turns dormant (resting) fibroblasts into myofibroblasts that generate an excessive amount of extracellular matrix (ECM) when stimulated by growth factors like TGF-*β* and platelet-derived growth factor (PDGF), as well as other cytokines. The intricate mechanisms of development entail the interaction of cytokines, signalling pathways and inflammatory cells that sustain an atypical cycle of damage and repair, ultimately resulting in the pathological accumulation of fibrous tissue and the impairment of normal organ function [254,256]. SCFAs directly and indirectly play an important role in pathophysiology of fibrosis by mediating these chemokines and cytokines.

Ischaemia is a restricted blood supply that causes oxygen insufficiency in tissues, muscle groups or organs, resulting in tissue damage or malfunction. Literature has established an association of SCFAs, particularly succinate, with IBDs leading to fibrosis. Studies on both humans and mice reported the increased succinate concentration in faeces of IBD subjects/patients [82,83, 86, 254, 257]. Ischaemic conditions cause cardiac, kidney and liver fibrosis by inducing necroptosis, hypoxia and accumulation of ECM, which release damage-associated molecular patterns (DAMPs) such as HMGB1, DNA and ATP. These DAMPs activate TLRs, causing pro-inflammatory chemokine and cytokine expression, and the ECM, namely types I and III collagen, to be released as a result, leading to fibrosis in these organs [241,258,261]. SCFAs, particularly succinate, accumulate during ischaemia and are promptly metabolized at ischaemic reperfusion, causing ROS evolution and cell death (necroptosis), driving towards the process of fibrosis development [262,264].

Dysbiosis, a condition where gut bacteria are unable to produce sufficient soluble fatty acids (SCFAs), can lead to inflammation and impaired immunity [239]. This is particularly true in cases of MS, a chronic inflammatory disease characterized by demyelination, gliosis, neuro-axonal damage and inflammation [79]. Macrophages, major WBCs, trigger inflammation in these conditions [186]. Article by Wrigley-Carr *et al.* [265] has reviewed in detail the imbalance (dysbiosis) in the gastrointestinal microbiome metabolites, particularly SCFAs, that occur in patients with CF, a hereditary illness that affects several organs. To fully comprehend SCFAs function in multifaceted CF illness and its potential therapeutic advantages, rigorous research is required [265]. Other organ fibrosis related to dysbiosis of metabolites (SCFAs) is discussed in here [255,266,271].

When tissue is damaged, fibroblasts become activated and develop into myofibroblasts. Smooth muscle cells, resident fibroblasts and epithelial cells transitioning through the epithelial-to-mesenchymal transition are some of the progenitor sources involved in this activation [272]. In stress fibres, myofibroblasts exhibit *α*-smooth muscle actin, which stimulates the release of ECM constituents like proteoglycans, fibronectin (FN), elastin and collagen types I, III, IV, V and VI [273]. FN promotes wound healing by facilitating cell adhesion and migration [274]. It also binds TGF-*β*, VEGF and PDGF, which influence myofibroblast development [275]. Fibrillin and LTBPs generate microfibrils that integrate TGF-*β* into the ECM, promoting tissue integrity and healing [276].

TGF-*β* regulates several tissue healing molecules, such as FNs, elastin, fibrillin, LTBP, CCN2, tenascin, proteoglycans, ECM components, collagen and activating SMAD signalling pathways to increase ECM gene expression. TNF-*α*, IL-1*β* and HGF are among the cytokines that impact fibroblast activity and stimulate ECM production [277]. It regulates their synthesis, secretion and activity, which all contribute substantially to wound healing, and any dysregulation in this can lead to profibrotic conditions and fibrosis [277].

Butyrate is the main SCFA that is substantially associated with enhanced TGF-*β* expression in epithelial cells. This activation causes increased TGF-*β* gene activity and protein levels, which are essential for intestinal homeostasis [278,279]. Table 2 describes the role of TGF in various repair molecules. Germ-free mice inoculated with gut bacteria that produce SCFAs have increased TGF-*β* expression and regulatory T cells [279,280]. TGF-*β* affects macrophage function by regulating differentiation and cytokine production. It can promote inflammation (M1 macrophage) or resolve responses through immunosuppressive activities (M2 macrophage) [256]. However, excessive or dysregulated TGF-*β* signalling can lead to persistent inflammation (main factor in fibrosis) and cancer development [281]. Dysbiosis, which causes reduced SCFA synthesis, can impair normal TGF-*β*-mediated homeostasis, potentially leading to the development of inflammatory illnesses and cancer over time [239,282]. Perpetual stimulation from TGF-*β* inhibits myofibroblast apoptosis, leading to an excessive buildup of collagen [283,284]. Unregulated production of cytokines, such as TNF-*α* and IL-1*β*, increases the production and deposition of ECM [285]. Also, anomalous ECM stiffening fuels the activation of myofibroblasts, ultimately leading to the formation of fibrotic tissue [286].

**Table 2.** Interaction of TGF-*β* in various repair molecules

Repair molecule	Role of TGF-*β*	Interaction with repair molecule	Receptors	Function	References
Collagens	Promotes synthesis of types I and III	Increases collagen production	TGF-*β* receptors	Stimulates ECM synthesis and myofibroblast differentiation	[342]
FNs	Induces alternative splicing of FN	Binds LTBPs, enhances FN matrix	TGF-*β* receptors	Necessary for myofibroblast differentiation, stabilizes latent TGF-*β* complex	[343,344]
Elastin	Stimulates elastin production	Enhances elastin expression	TGF-*β* receptors	Increases ECM elasticity and structural integrity	[345,346]
Fibrillin and LTBP	Targets growth factors to ECM	Stores and activates TGF-*β*	TGF-*β* receptors	Regulates TGF-*β* bioactivity, supports ECM integrity	[347]
CCN2 (CTGF)	Induces CCN2 expression	Synergizes with TGF-*β*	TGF-*β* receptors	Enhances fibrosis and myofibroblast activation	[348]
Tenascin	Supports stromal cell migration	Prevents apoptosis	TGF-*β* receptors	Promotes myofibroblast recruitment and persistence	[349]
Proteoglycans	Facilitates ECM assembly	Incorporates TGF-*β* into ECM	TGF-*β* receptors, CD44	Modulates myofibroblast activation and persistence through HA and CD44 interaction	[344]
ECM components	Modulates stiffness and cross-linking	Cross-links with ECM proteins	TGF-*β* receptors, integrins	Promotes ECM protein cross-linking, enhances tissue stiffness	[279,350]

Other anti-inflammatory and antifibrotic effects of SCFAs are associated with their ability to regulate various organ-associated immune cells, i.e. macrophages, DCs, neutrophils, eosinophils, T cells, B lymphocytes and ILCs, by mediating anti-inflammatory cytokines and chemokines through various pathways (MAPKs), as well as suppressing pro-inflammatory mediators by histone acetylation (HDAC inhibition). This plays a role in the antifibrotic role via regulated inflammation, wound healing and tissue injury [22].

**Fig. 4.**
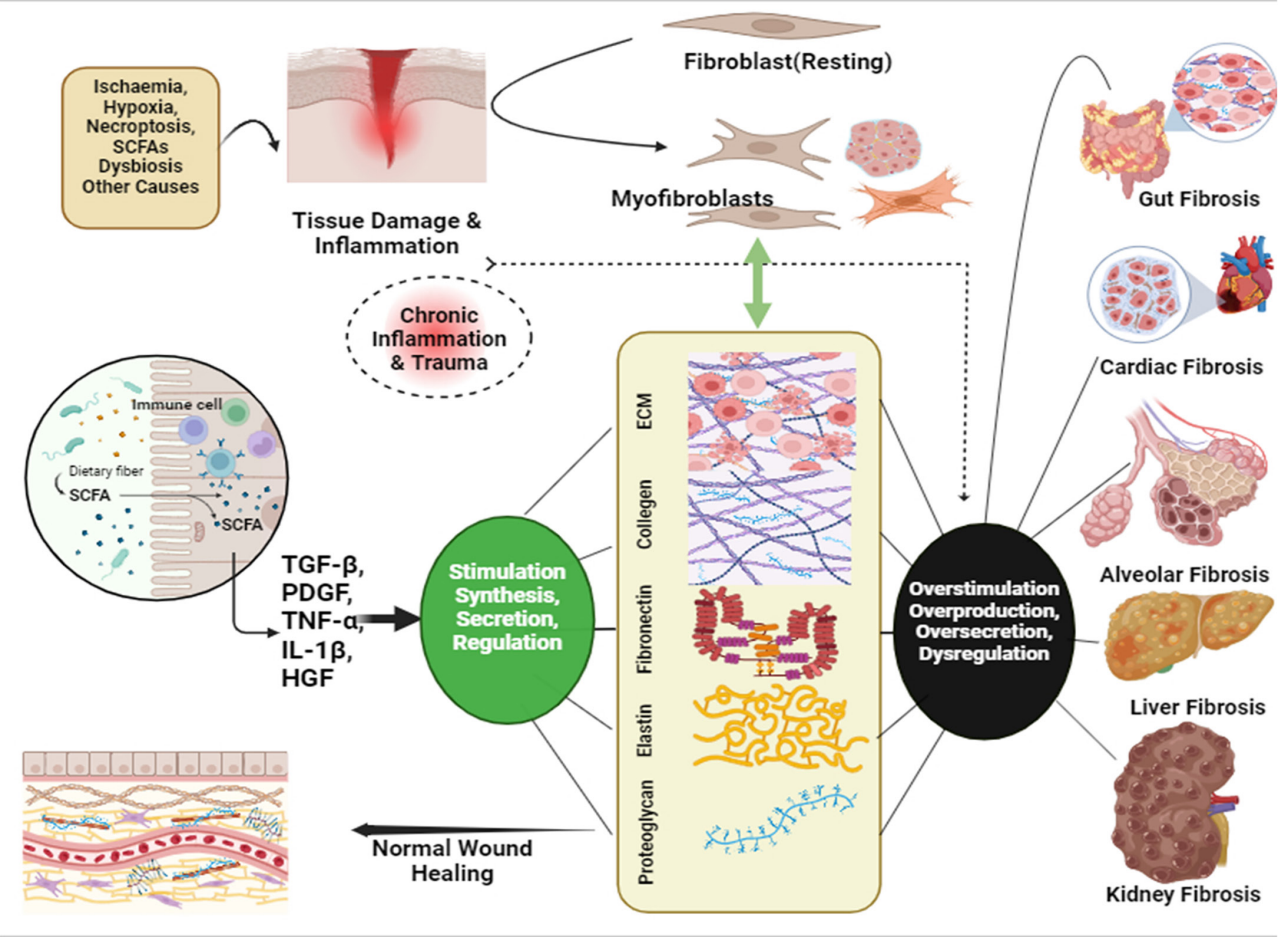
SCFAs mediated cytokines and chemokines which stimulate synthesis, secretion and regulation of different wound healing mediators and proteins. Dysregulation in this wound healing process causes the over-stimulation and overproduction/deposition of these mediators, which can cause organ fibrosis. Dysregulation can be a result of perpetuating pro-inflammatory TGF-*β* by SCFAs, chronic inflammation and trauma.

SCFAs, such as butyrate and valproate, are shown to inhibit fibroblast differentiation and death through various pathways. These mechanisms include inhibiting TGF-*β*1 (excessive TGF-*β*), enhancing H3K27 acetylation, suppressing Akt (protein kinase B) expression, regulating macrophage differentiation and inhibiting HDAC3 [287]. Collectively, these actions have a therapeutic impact on pulmonary fibrosis. Additionally, SCFAs mitigate lung fibrosis by suppressing both viral and inflammatory responses through modulation of the immune system, as discussed in the following reviews [287,292]. Fig. 4 provides the visual representation of SCFA-stimulated cytokines and chemokines (TGF-*β*, ECM components) regulation of fibrotic conditions.

Studies have demonstrated that treating rats with forsythiaside A, a chemical produced from plant fibre that optimizes the gut microbiota to promote SCFAs, improves fibrosis of the liver in rats by lowering oxidative stress and inflammation, changing the gut microbiota and raising levels of SCFA. Moreover, Forsythiae fructus’s main lignan component, phillygenin (PHI), possesses potent antioxidant, hepatoprotective and anti-inflammatory properties [240,293]. While the study does not directly quantify SCFAs, the reported modulation of gut microbiota by *Phyllostachys nigra* polysaccharide may indirectly alter SCFA production, thereby contributing to improvements in glycolipid metabolism and potentially influencing inflammatory pathways [294]. Wang *et al.* discovered that PHI increases the synthesis of SCFAs in mouse intestines, consequently reducing carbon tetrachloride-induced liver fibrosis [295]. A review by Pohl *et al.* [296] assessed 17 researches, including 2 human and 15 murine models, and found 58 indicators of liver damage and 26 distinct measures of permeability. Although a meta-analysis was not possible due to the variety of study designs, SCFA supplementation via oral and enteral routes, butyrate, probiotics and prebiotics consistently demonstrated improved permeability and a significant reduction in liver injury, including fibrosis. Collectively, this data highlight SCFAs’ therapeutic promise in treating liver fibrosis through anti-inflammatory, antioxidant and gut microbiota-modulating properties.

## A delicate line between aggravation and resolution

While SCFAs’ anti-inflammatory effects are well established, their ability to exacerbate inflammation and fibrosis is less explored. This study vacuum may be due to a trend-following attitude in academia, where the dominant narrative of SCFAs as therapeutic agents might overshadow enquiries addressing their potential, inherently pro-inflammatory properties. In the context of academic trend-following and marketing bias, the emphasis on the positive benefits of SCFAs may have disguised their pro-inflammatory properties. The wellness industry’s growing interest in science-based health solutions has driven consumer demand for health-promoting products and concepts thought to improve gut health, immunity and general well-being. This consumer interest has sometimes influenced the trajectory of scientific research, with studies highlighting good effects being more easily marketable and funded. Also, due to resource constraints, the negative consequences of these metabolites, such as their pro-inflammatory potential under specific situations, have remained unknown [297,301].

### Inherent nature

The pro-inflammatory nature and association with pro-inflammatory diseases of SCFAs, particularly succinate, have been elucidated in many studies and literature reviews, demonstrating that not all SCFAs are inherently anti-inflammatory [302]. A study reported by Chouchani and co-authors [303] showed that the accumulation of cytosolic succinate leads to ROS generation and inflammation, a significant marker in ischaemia. Tannahill *et al.* [304] presented a study showing that macrophage activation by succinate produced pro-inflammatory cytokines IL-1*β* via HIF-1*α* transcription. Data from Littlewood-Evans and colleagues [305] presented similar results where succinate through GPR91 produced pro-inflammation (IL-1*β*) in RA. T cells under the influence of SCFAs turn into effector cells (Th17, Th1 and Tc), which either lead to pro-inflammation or anti-inflammation depending on the cytokine conditions. CD4+ T cells isolated from mice were treated with SCFAs (C2), resulting in the direct promotion of the pro-inflammatory nature of T cells, as concluded by Park *et al.* [306] in their experimental results.

GPR43 (FFAR2) activates a pathway involving the *β*-arrestin-2 subunit, resulting in the release of anti-inflammatory cytokines [61,307]. The results of Sina *et al.* [97], Maslowski *et al.* [98] and Masui *et al.* [91] experiments are contrasting, highlighting the pleiotropic nature of SCFA receptors. This suggests that SCFAs can lead to either pro-inflammation or the resolution of inflammation.

### Investigation methodologies

This current discussion pertaining to the dual roles of SCFAs in the gut may also be influenced by constraints such as study samples primarily being faeces, which may not always be reliable due to the presence of impurities and nutrient depletion [308,309]. Furthermore, epidemiological studies highlight the potentially negative impact of SCFAs in the body [310].

### Cell-type-specific responses

The response to SCFAs varies significantly among cell types. SCFAs can reduce pro-inflammatory responses in epithelial cells and macrophages, increasing gut health and lowering fibrosis [22]. However, they can also stimulate other immune cells, such as T cells, resulting in a pro-inflammatory state. Park *et al.* [306] showed the inflammatory nature of T cells in response to treatment with C2 SCFA, whereas antigen-presenting cells in microglia upon treatment with SCFAs (C2, 3 and 4) showed a suppressive effect on inflammatory mediators by increased expression of IL-10 and HIF-1*α*. The delicate intersection between the induction of inflammation and its resolution is also dependent on the presence/expression level of receptors on cells. Activated GPR41 and GPR43 receptors by SCFAs in mice epithelial cells promote inflammation [57], while GPR43 receptor-devoid cells showed opposite results in DSS-induced colitis mice [97]. Similarly, SCFAs, particularly butyrate, in one study, regulate NF*κ*B pathway via ROS and neddylation and suppress inflammation. While other studies showed that butyrate imparts ROS-induced apoptosis and inhibition of cell cycle. Cancerous cell lines behave differently when activated by SCFAs, contrary to normal cells. Propionate induces antioxidant mechanisms in cancerous cells as opposed to butyrate. This might be due to the Warburg effect, in which these cells divert energy production from common pathways, rather than imply anaerobic high sugar levels and resultant lactate production [311]. SCFAs, particularly butyrate, which is a primary energy provider, accumulate and, being HDAC inhibitors, cause upregulation of varied genes [312]. The role of SCFAs in young rats was shown to be deteriorating and injury-induced compared to matured ones. Furthermore, SCFAs behave differently when administered orally versus systematically. For example, considering the study of Berndt *et al.*, DSS-induced colitis became more inflammopathic with adverse severity in rats when butyrate was fed through drinking water. On the other hand, IP injection of butyrate reversed the effects of DSS-induced colitis [132].

The role of acetate in the brain of mice with a high-fat-based diet-induced obesity is to activate the parasympathetic nervous system (PNS), leading to insulin resistance by dysregulating metabolism. Similarly, acetate also inhibits insulin release in beta cells of the pancreas through FFAR2 activation. However, diabetic mice exhibit glucose intake and insulin secretion when there are high levels of acetate in the pancreas and cytoplasm [76,313]. Contradictory results are also observed regarding the therapeutic effects of SCFAs on cancer [314]. The literature does not provide much information on such studies in humans. Succinate derived from diet was found to be regulating insulin plus glucose homeostasis in generic type as opposed to mutant mice [315].

Considering the above-mentioned data, most of these studies have been conducted on mice, and we can conclude that SCFAs exhibit a dual nature, acting as both heroes and villains in the realm of host immunometabolic physiology. This duality of SCFAs can also be explained by the limited but conflicting data obtained from *in vitro* studies, direct stimulations and *in vivo* receptor gene targeting, suggesting that SCFAs serve not only as nutritional substrates but also as signalling molecules.

### Influence of pathogens

Pathogenic micro-organisms can significantly alter SCFAs behaviour, causing inflammation and fibrosis. Bacteria like *Salmonella* and *Shigella* can migrate through the gut lining, reducing SCFA levels, which triggers a feedback loop, allowing pathogens to colonize and develop in the gut environment. Chronic spontaneous urticaria is an inflammatory disease in which gut microbiota is altered and replaced with *Klebsiella pneumoniae* and *Roseburia hominis*. A study by Zhu *et al.* showed the elevated LPS by altered microbiota level suppressed the SCFAs level and facilitated inflammation [316]. Another study by Vinolo *et al.* [202] demonstrated suppression of LPS-induced inflammation by SCFAs (C2, 3, 4). Both studies suggest the different results are due to the presence and interaction of pathogenic bacteria with SCFAs. This might partly be due to the possibility of reduction in beneficial SCFA-producing bacteria in the presence of pathogenic bacteria that lead to inflammation in the former study.

**Fig. 5.**
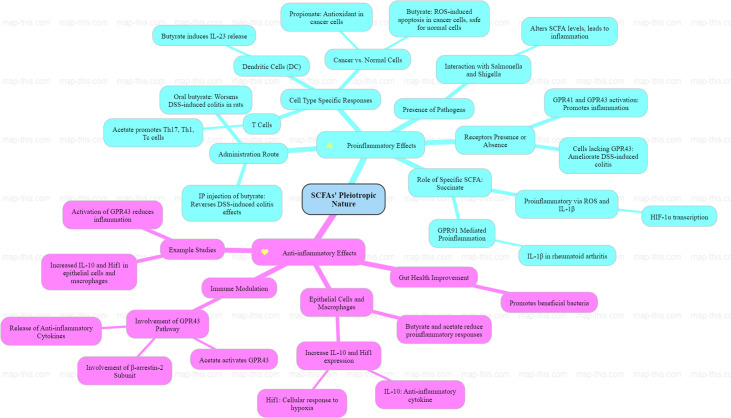
A contrasting comparison and factors which influence the delicate intersection between aggravation and resolution of inflammation and fibrosis. (Designed using mind-this.com; can be accessed online at https://shorturl.at/UpbLX) [351].

The above given data and studies compel us to conclude that SCFAs play pleiotropic effects depending on the cell type, age, mode of infusion, dosage, variations in study methods and inherent properties of individual SCFAs (Fig. 5). This negates the common notion of only beneficial effects of these gut microbiota metabolites, thus emphasizing the necessity of further research on this complex nature of these molecules.

## Conclusion

SCFAs are formidable regulators of the host immunological response, effectively allowing communication between the intestines and other organs. Their ambivalent impact originates largely from the metabolic and metagenomic changes they cause, as well as the signalling pathways linked to FFARs, which regulate both the adaptive and innate immune systems in the gut and beyond. Recent research has highlighted the dual nature (pleiotropic effects) of SCFAs, indicating their role in both pro-inflammatory and anti-inflammatory processes in acute and chronic inflammatory illnesses and infections. SCFAs have important roles in modifying immune responses and affecting inflammation, but their complicated mechanisms in pro- and anti-inflammatory processes, as well as fibrosis, are poorly understood. Traditional research, which largely uses rodent models, has limits in transferring results to people due to species-specific characteristics. A more human-centric research approach is needed to fully understand the accurateness and nature of SCFAs in various scenarios. Three advanced models are being developed to improve the specificity of SCFA interaction research: organoids, gut-on-chip systems and humanized mice. Organoids generated from human stem cells closely resemble human tissue architecture and biological responses [317]. Gut-on-chip models mimic the intestinal environment, enabling comprehensive investigation of microbiota and SCFA effects under controlled settings [318]. Humanized mice, which are bred to have human-like immune systems, provide a more precise platform for researching human immune responses and creating treatments [319]. These models are hoped to reflect substantial advances in SCFA research, offering more accurate and human-relevant insights. Also, combining physiologically realistic culture systems or animal models with controlled SCFA dosing, diverse cell types/receptor assays and omics-based readouts will most accurately reveal SCFAs’ true *in vivo* roles, including the delicate balance between their pro- versus anti-inflammatory effects.

## Abbreviations

BCFAs: branched-chain fatty acids
CD: Crohn’s disease
CeD: coeliac disease
CF: cystic fibrosis
DCs: dendritic cells
ECM: extracellular matrix
ERK: extracellular signal-regulated kinase
FFAR: free fatty acid receptor
FN: fibronectin
GPCRs: G-protein-coupled receptors
HDACs: histone deacetylases
IBD: inflammatory bowel disease
ILCs: innate lymphoid cells
iNOS: inducible NO synthase
IP3: inositol 1,4,5-trisphosphate
MCT: monocarboxylate transporter
MS: multiple sclerosis
MUFA: monounsaturated long-chain fatty acid
NAFLD: non-alcoholic fatty liver disease
NK: natural killer
PAMPs: pathogen-associated molecular patterns
PDGF: platelet-derived growth factor
PHI: phillygenin
PKC: protein kinase C
PRRs: pattern recognition receptors
RA: rheumatoid arthritis
ROS: reactive oxygen species
SCFAs: short-chain fatty acids
SIRS: systemic inflammatory response syndrome
SLE: systemic lupus erythematosus
T1D: type 1 diabetes
TGF-*β*: transforming growth factor-beta
TLRs: toll-like receptors
TMA: trimethylamine
TMAO: trimethylamine-*N*-oxide
UC: ulcerative colitis
VSM: vascular smooth muscle
WBCs: white blood cells
